# Metallopeptidase inhibitor 1 (TIMP‐1) promotes receptor tyrosine kinase c‐Kit signaling in colorectal cancer

**DOI:** 10.1002/1878-0261.12575

**Published:** 2019-10-24

**Authors:** Cathrine Nordgaard, Sophia Doll, Ana Laura de Souza Almeida Matos, Mikkel Høeberg, Julhash Uddin Kazi, Stine Friis, Jan Stenvang, Lars Rönnstrand, Matthias Mann, José Manuel Afonso Moreira

**Affiliations:** ^1^ Department of Drug Design and Pharmacology Faculty of Health and Medical Sciences University of Copenhagen Denmark; ^2^ Department of Proteomics and Signal Transduction Max Planck Institute of Biochemistry Martinsried Germany; ^3^ Novo Nordisk Foundation Center for Protein Research Faculty of Health Sciences University of Copenhagen Denmark; ^4^ Division of Translational Cancer Research and Lund Stem Cell Center Lund University Sweden; ^5^ Division of Oncology Skåne University Hospital Lund Sweden

**Keywords:** colorectal cancer, c‐Kit, cetuximab, predictive biomarkers, omics, invasion

## Abstract

Colorectal cancer (CRC) is the third most prevalent cancer worldwide causing an estimated 700 000 deaths annually. Different types of treatment are available for patients with advanced metastatic colorectal cancer, including targeted biological agents, such as cetuximab, a monoclonal antibody that targets EGFR. We have previously reported a study indicating multiple levels of interaction between metallopeptidase inhibitor 1 (TIMP‐1) and the epidermal growth factor (EGF) signaling axis, which could explain how TIMP‐1 levels can affect the antitumor effects of EGFR inhibitors. We also reported an association between TIMP‐1‐mediated cell invasive behavior and *KRAS* status. To gain insight into the molecular mechanisms underlying the effects of TIMP‐1 in CRC, we examined by transcriptomics, proteomics, and kinase activity profiling a matched pair of isogenic human CRC isogenic DLD‐1 CRC cell clones, bearing either an hemizygous *KRAS* wild‐type allele or KRAS G13D mutant allele, exposed, or not, to TIMP‐1. Omics analysis of the two cell lines identified the receptor tyrosine kinase c‐Kit, a proto‐oncogene that can modulate cell proliferation and invasion in CRC, as a target for TIMP‐1. We found that exposure of DLD‐1 CRC cells to exogenously added TIMP‐1 promoted phosphorylation of c‐Kit, indicative of a stimulatory effect of TIMP‐1 on the c‐Kit signaling axis. In addition, TIMP‐1 inhibited c‐Kit shedding in CRC cells grown in the presence of exogenous TIMP‐1. Given the regulatory roles that c‐Kit plays in cell proliferation and migration, and the realization that c‐Kit is an important oncogene in CRC, it is likely that some of the biological effects of TIMP‐1 overexpression in CRC may be exerted through its effect on c‐Kit signaling.

## Graphical abstract

We identified a proto‐oncogene, c‐Kit, as a potential mediator of metallopeptidase inhibitor 1 (TIMP‐1) function in colorectal cancer (CRC) cells. Given the role that c‐Kit plays in regulation of cell proliferation and migration, it is likely that some of the effects of TIMP‐1 overexpression in CRC may be exerted through its effect on c‐Kit signaling.

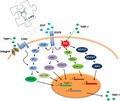

**Abbreviations**
CE: cellular extracts
CM: conditioned media
CRC: colorectal cancer
DAPI: 4′,6‐diamidino‐2‐phenylindole
ECM: extracellular matrix
EGF: epidermal growth factor
MAPK: mitogen‐activated protein kinase
MMP: matrix metalloproteinase
MS: mass spectrometry
PLA: proximity ligation assay
RTK: receptor tyrosine kinase
STK: serine/threonine kinase
TIMP‐1: metallopeptidase inhibitor 1
TK: tyrosine kinase
TRIM59: tripartite motif‐containing 59
Y2H: yeast two‐hybrid

## 1 Introduction

Metallopeptidase Inhibitor 1 (TIMP‐1) was originally discovered as a serum protein responsible for collagenase inhibition and growth of erythroid progenitor cells and is one of the four known endogenous human metallopeptidase inhibitors (TIMP‐1 through TIMP‐4). TIMP‐1 is expressed in various tissues, including colon, and as the name suggests, it inhibits matrix metalloproteinases (MMPs), a family of peptidases regulating extracellular matrix (ECM) turnover. TIMPs and MMPs regulate tumor invasion, migration, and progression through tissue remodeling. Accordingly, one would expect TIMP‐1 to exert mainly a beneficial effect on cancer outcomes, by inhibiting remodeling of the ECM and therefore blocking invasion and metastasis. However, regulatory effects on cell proliferation and survival have also been reported for TIMP‐1, with both positive and negative effects on tumor growth and progression reported (Hornebeck *et al.*, 2005). Available evidence suggests TIMP‐1 to work in a dual way, regulating cellular functions by both MMP‐dependent and MMP‐independent mechanisms. Structurally, TIMP‐1 consists of two distinct and independent structural domains: an N‐terminal domain, spanning the MMP‐inhibitory activity, and a C‐terminal domain thought to hold some of the MMP‐independent cytokine‐like activities of TIMP‐1 (Chesler *et al.*, 1995; Gasson *et al.*, 1985; Herszenyi *et al.*, 2012; Stetler‐Stevenson, 2008). The localization of TIMP‐1 may be crucial to explain its dual effects: Extracellular TIMP‐1 confined to the ECM prevents metastasis by inhibiting nearby MMPs, whereas intracellular TIMP‐1 promotes cancer cell proliferation and survival by effecting different signaling pathways. These different effects may also be modulated by different interaction partners. TIMP‐1 interacts with a complex of the tetraspanin cluster of differentiation 63 (CD63) and integrin β1 at the cell membrane (Jung *et al.*, 2006), and CD63 down‐regulation decreases TIMP‐1 presence at the cell surface and reduces cell survival in breast cancer cells. The TIMP‐1–CD63–integrin β1 complex can also stimulate Src phosphorylation, followed by activation of yes‐associated protein and transcriptional co‐activator with PDZ binding domain, which in turn promote cancer cell proliferation (Ando *et al.*, 2018). TIMP‐1 also interacts with a complex of the cell surface glycoprotein cluster of differentiation 44 and pro‐MMP‐9, and these mediate TIMP‐1′s antiapoptotic effects in erythroid cells (Lambert *et al.*, 2009). In addition, TIMP‐1 can also up‐regulate *RAS* and *MAPK* activity, possibly through activation of receptor tyrosine kinases (RTKs) (Akahane *et al.*, 2004; Wang *et al.*, 2002; Yamashita *et al.*, 1996).

Recently, we found that TIMP‐1 plasma levels predicted benefit from EGFR‐inhibition therapy in *KRAS*‐mutated metastatic colorectal cancer patients. These results were supported by data from cellular models showing that exposure of colorectal cancer (CRC) cells to exogenously added recombinant TIMP‐1 promoted a more aggressive behavior in these cells, specifically in *KRAS*‐mutated cells (Tarpgaard *et al.*, 2016). We added recombinant TIMP‐1 to a matched set of isogenic DLD‐1 CRC cell clones, in which either the endogenous *KRAS* wt allele (from here on referred to as DLD‐1 G13D cell line) or the *KRAS*(G13D) mutant allele (from here on referred to as DLD‐1 wt cell line) has been inactivated by targeted homologous recombination (Yun *et al.*, 2009), respectively. Whereas the DLD‐1 wt cells showed no significant difference in invasive potential, DLD‐1 G13D cells responded to the presence of TIMP‐1 in the growth medium by becoming significantly more invasive (Tarpgaard *et al.*, 2016).

To address the effect of TIMP‐1 on the invasive potential of CRC cells, we took a three‐pronged approach: We performed a comparative proteomic analysis of the DLD‐1 G13D and DLD‐1 wt CRC cells to identify differentially expressed proteins. We also analyzed the transcriptome of DLD‐1 G13D and DLD‐1 wt cells at baseline and following exposure to TIMP‐1 to identify changes caused by exposure to TIMP‐1. Finally, we performed a broad kinase activity assay study of the DLD‐1 G13D and DLD‐1 wt cells exposed to TIMP‐1, to identify differential effects on RTKs in these cells. As a result, we identified the RTK c‐Kit as a target of TIMP‐1 in these cells. *KIT* encodes the human homolog of the proto‐oncogene c‐Kit, the cellular homolog of the transforming oncogene of the Hardy–Zuckerman 4 feline sarcoma virus (Yarden *et al.*, 1987). c‐Kit is a RTK, and aberrant expression or mutational activation of c‐Kit is associated with a variety of diseases in humans, including cancer (Heinrich *et al.*, 2002; Lennartsson *et al.*, 2005). We found that TIMP‐1 increased phosphorylation of c‐Kit, as well as corresponding targets, and inhibited c‐Kit shedding. In summary, our data provide some further mechanistic insight into the effects of TIMP‐1 on CRC cells.

## 2 Materials and Methods

### 2.1 Cell culture

A set of isogenic colorectal adenocarcinoma cell lines, derived from the DLD‐1 CRC line by targeted homologous recombination of the *KRAS* locus, were used in this study (Yun *et al.*, 2009): The *KRAS*‐mutated (KRAS^G13D/−^) DLD‐1 cell line (referred to as DLD‐1 G13D) was generated by knockout of the wild‐type *KRAS* allele in heterozygous DLD‐1 parental cells (KRAS^G13D/+^), whereas the *KRAS* wild‐type (KRAS^+/−^) DLD‐1 cell line (referred to as DLD‐1 wt) was generated by knockout of the mutant *KRAS* allele, respectively. The cell lines were kindly provided by Bert Vogelstein (Johns Hopkins University, USA). All cell lines were grown under sterile conditions at 37 °C and 5% CO_2_ in McCoy’s 5A medium (Invitrogen, Carlsbad, CA, USA). Growth media were supplemented with 10% FBS (Invitrogen), unless stated otherwise. To investigate the role of TIMP‐1, cells were stimulated with 1 or 5 µg·mL^−1^ N‐glycosylated recombinant His_6_‐tagged human TIMP‐1, as described (Vinther *et al.*, 2014). As control, cells were stimulated with vehicle (PBS) or with an equivalent amount of BSA, as appropriate (Roche Diagnostics, Basel, Switzerland). The murine pro‐B cell line Ba/F3 was maintained in RPMI 1640 medium supplemented with 10% heat‐inactivated FBS and 10 ng·mL^−1^ recombinant murine IL‐3 (Prospec Tany, Rehovot, Israel) (Pedersen *et al.*, 2009). The identity of all cell lines used was verified by short tandem repeat loci profiling (IdentiCell, Aarhus, Denmark).

### 2.2 Western blot analysis

For protein extractions, we used the Mammalian Protein Extraction Reagent (M‐PER; Thermo Fisher Scientific, Waltham, MA, USA) supplemented with Pierce Protease and Phosphatase Inhibitor Mini Tablets (Thermo Fisher Scientific). The lysates were centrifuged at 14 000 ***g*** for 10 min to remove cell debris, and protein concentrations were determined using Thermo Scientific Pierce BCA Protein Assay Kit (Thermo Fisher Scientific). The samples were diluted with Laemmli sample buffer (Sigma‐Aldrich, St. Louis, MI, USA) to contain ~ 20 µg protein in 25 µL total volume or 15 µg protein in 15 µL total volume, depending on the use of either 10‐ or 15‐well gels (4–15% Mini‐PROTEAN^®^ TGC™ gel; Bio‐Rad, Hercules, CA, USA). Samples were incubated at 70 °C for 10 min prior to loading. The protein samples were resolved on a gel using Bio‐Rad SDS System (Bio‐Rad) and blotted onto a 0.2‐µm nitrocellulose membrane (Trans‐Blot^®^ Turbo™ Midi Nitrocellulose Transfer Pack; Bio‐Rad). Membranes were blocked with 5% skim milk powder (Merck Life Sciences, Darmstadt, Germany) or 5% BSA fraction V (Roche Diagnostics) in TBS‐T, before being incubated with primary antibodies followed by a horseradish peroxidase ‐conjugated secondary antibody in blocking buffer. Protein bands were developed using either Clarity Western ECL Substrate (Bio‐Rad) or Amersham ECL Select Western Blotting Detection Reagent (GE Healthcare Life Sciences, Piscataway, NJ, USA), as appropriate. Images were acquired with UVP BioSpectrum Imaging System (UVP, Upland, CA, USA), and bands were quantified using the software (imagej version 1.49, U. S. National Institutes of Health, Bethesda, MD, USA).

### 2.3 Immunostaining

Cells were washed, trypsinized, and then fixed with 4% buffered formalin (Sigma‐Aldrich). Following extensive washings with PBS, cells were centrifuged to form a pellet, which was kept in 70% EtOH (Sigma‐Aldrich) until embedded in paraffin. Before use, embedded cells were freshly cut into 3‐µm sections, deparaffinized in xylene, and rehydrated through an alcohol gradient. Sections were exposed to heat‐induced antigen retrieval and placed in EnVision™ FLEX Target Retrieval Solution, low pH (Dako, Glostrup, Denmark) diluted 1 : 50 in MilliQ H_2_O and boiled. For some of the experiments, cells were grown on coverslips or in Permanox chamber slides (Lab‐Tek Chamber Slide System; Thermo Fisher Scientific) in order to avoid trypsin treatment or cell scraping. The surfaces were coated with poly‐l‐lysine (Sigma‐Aldrich) prior to seeding. Cells were washed twice with ice‐cold PBS prior to 20 min of fixation with 4% buffered formalin. Cells were permeabilized with 0.05% Triton X‐100 in Tris‐buffered saline (TBS; 20 mm Tris pH 7.6, 150 mm NaCl) for 5 min at RT and washed with 1× TBS. Coverslips and chamber slides were kept in humidity chambers during all incubations. Prior to incubation of primary antibody, sections were blocked with 25% BSA in TBS before incubation with primary antibodies. Sections were then incubated with secondary Alexa Fluor antibodies (Invitrogen) diluted in 25% BSA in TBS. Slides were mounted with ProLong Gold Antifade Reagent with 4',6‐diamidino‐2‐phenylindole (DAPI; Invitrogen) and examined by confocal microscopy. All antibodies used in this study, and the dilutions used, are listed in Table S1.

### 2.4 Proximity ligation assay

Proximity ligation assay (PLA) (Duolink; Olink Bioscience, Uppsala, Sweden) was performed according to the manufacturer’s instructions. Briefly, cell pellets mounted on glass slides were blocked with Duolink blocking buffer for 30 min at 37 °C in humidity chamber. Blocking buffer was tapped off, and primary antibodies were diluted in Duolink Antibody Diluent (TIMP‐1: 1 : 4000 and c‐Kit: 1 : 250). Slides were mounted with cover plates, placed in cassettes or in humidity chamber (for embedded cells vs chamber slides), then incubated with either one of the antibodies as control or both, and left to incubate at 4 °C overnight. The following day slides were washed with wash buffer A (Duolink) 2 × 5 min before released from cassettes and incubated for 1 h at 37 °C with both PLA probes diluted 1 : 5 in Antibody Diluent. PLA probes were tapped off, and slides were washed twice for 5 min with wash buffer A. Ligation stock was diluted 1 : 5 in MilliQ water, and ligase was diluted in the mixture 1 : 40. Ligation–ligase was added to slides, which were then incubated 30 min at 37 °C and then tapped off. Sections were washed 2 × 2 min with wash buffer A. Amplification stock was diluted 1 : 5, and polymerase was diluted 1 : 80 in this mixture. Amplification–polymerase mixture was added to the slides followed by incubation for 100 min at 37 °C. Amplification‐polymerase was tapped off, and slides were washed once either for 10 min in wash buffer B or for 5 min in wash buffer B, followed by 15‐min incubation with Atto488 Phalloidin (Sigma‐Aldrich) diluted 1 : 100 in wash buffer B, and subsequently washed with wash buffer B for 10 min. Slides were washed 1 min in 0.01× wash buffer B and dried in the dark prior to mounting with Duolink In Situ Mounting Medium with DAPI. Edges were sealed with nail polish, and slides were imaged with confocal microscopy.

### 2.5 Kinase activity assay

DLD‐1 wt and DLD‐1 G13D cells were serum‐starved for 4 h prior to 15‐, 30‐, and 120‐min stimulations with 5 µg·mL^−1^ TIMP‐1 or vehicle. Cells were washed with 3× cold PBS and lysed with M‐PER lysis buffer. Protein concentrations were measured, and samples were diluted to equal concentration. Kinase activity assay was performed using PamChip tyrosine kinase (TK) and serine/threonine kinase (STK) arrays (PamGene, Hertogenbosch, the Netherlands). STK arrays consist of 140 ser/thr‐containing peptides. TK arrays consist of 144 peptides with known phosphorylation sites, representing 100 different proteins. Each peptide represents a 15‐amino‐acid sequence of which 13 residues are derived from a putative phosphorylation site present in human proteins. Peptide sequences are derived from literature and are a cognate target of one or multiple kinases. Activation status of represented kinases is derived from the phosphorylation levels of its combined cognate targets. Incubation, washing, dispensing of reagents, and imaging of the PamChip were performed automatically using the PamStation 12. PamGene’s Bionavigator software was used for image quantification, quality control, statistical analysis, visualization, and interpretation. Downstream data analysis was done using MetaCore pathway analysis software (Thomson Reuters, Toronto, Canada).

### 2.6 c‐Kit shedding ELISA kit

To measure TIMP‐1’s effect on shedding of c‐Kit to the media, c‐Kit (Soluble) (Human) ELISA Kit (Nordic Biosite, Copenhagen, Denmark) was employed. This kit measures the levels of soluble c‐Kit as readout for shedding of c‐Kit. The experiments were performed in accordance with the manufacturer’s instructions.

### 2.7 Gene expression assays

DLD‐1 wt and DLD‐1 G13D cells were cultured in the presence, or absence, of TIMP‐1 (5 µg·mL^−1^) for 4 h, washed thrice, and collected by trypsinization in three independent, consecutive (passages 28, 29, and 30) experiments. The RNeasy Mini Kit (Qiagen, Venlo, The Netherlands) was used to isolate RNA from cells as described in the manufacturer’s protocol. In order to optimize samples for gene expression assay, samples were treated with RQ1 RNase‐free DNase (Promega, Madison, Wisconsin, WI, USA); 1 µL DNase was used per µg RNA, samples were mixed with 1 µL reaction buffer, and RNase‐free water was added to a volume of 10 µL. Samples were incubated at 37 °C for 30 min before 1 µL of DNase stop solution was added followed by 10 min of incubation at 65 °C to terminate the reaction. Samples were stored at −80 °C before processing.

Samples were analyzed at the Multi‐Assay Core Facility at the Danish Technical University (Center for Biological Sequence Analysis, DTU) using Agilent arrays. Briefly, samples were labeled using Low Input Quick Amp Labeling Kits (One‐Color; Agilent Technologies, Santa Clara, CA, USA) in accordance with the manufacturer’s instructions. Arrays were processed and data were collected using Agilent Feature Extraction Software (Agilent Technologies). Data analysis was done using the partek genomics suite v7.0 software package (Partek, St. Louis, MO, USA). Results are presented as Log_2_FoldChange of DLD‐1 wt vs DLD‐1 G13D, and TIMP‐1‐treated vs TIMP‐1‐untreated cells, genes were considered significantly different expressed if *P*‐value < 0.0075 and Log2FoldChange>+/‐1. Multiple testing correction of *P*‐values was performed using the Benjamini–Hochberg method (Benjamini and Hochberg, 1995).

### 2.8 *In vitro* wound healing assay

DLD‐1 wt cells were plated at a density of 5 × 10^4^ cells/well in 2‐well silicone inserts (ibidi GmbH, Gräfelfing, Germany) and incubated at 37 °C for 24 h. Then, inserts were removed leaving a 500‐µm gap without any cells. Dishes were cultured for 24 h in McCoy’s 5A medium (Invitrogen), supplemented with 10% FBS (Invitrogen), and in the presence, or absence, of TIMP‐1 (5 µg·mL^−1^), imatinib mesylate (Sigma‐Aldrich), or both, and imaged for gap size measurement. Three nonoverlapping representative images from each of the gapped areas were acquired to estimate the relative migration of cells. Each condition was examined in triplicate experiments.

### 2.9 Mass spectrometry (MS)‐based proteomic analysis

Cells were processed according to the in stage tip sample preparation method (Kulak *et al.,* 2014). Briefly, 250 µL of the reducing and alkylating sodium deoxycholate buffer (PreOmics) was added to the samples before protein denaturation at 99 °C and 1500 *g* for 10 min. Samples were further solubilized using the bioruptor for 10 min and digested by trypsin and Lys‐C (1 : 100) overnight at 37 °C and up to 2000 *g*. Following peptide desalting using SDB‐RPS, samples were measured on an quadrupole–Orbitrap mass spectrometer (Scheltema *et al.,* 2014; Kelstrup *et al.,* 2014) (Q Exactive HF; Thermo Fisher Scientific, Rockford, IL, USA) coupled to an EASYnLC 1200 ultra‐high‐pressure system (Thermo Fisher Scientific). About 0.5 µg of peptides were loaded on a 45‐cm HPLC column (75 μm inner diameter; in‐house‐packed using ReproSil‐Pur C18‐AQ 1.9 µm silica beads; Dr. Maisch GmbH, Ammerbuch, Germany). Peptides were separated using a linear 100 min gradient from 3% to 23% B in 82 min and stepped up to 40% in 8 min at 350 nL per min where solvent A was 0.1% formic acid in water and solvent B was 80% acetonitrile and 0.1% formic acid in water. Column temperature was kept at 60 °C. The mass spectrometer was operated in ‘top‐15’ data‐dependent mode, collecting MS spectra in the Orbitrap mass analyzer (60 000 resolution, 300–1650 *m/z* range) with a maximum ion injection time of 25 ms and an automatic gain control (AGC) target of 3E6. The most intense ions from the full scan were isolated with a width of 1.4 *m/z*. Following higher‐energy collisional dissociation with a normalized collision energy of 27%, MS/MS spectra were collected in the Orbitrap (15 000 resolution) with a maximum ion injection time of 28 ms and an AGC target of 1E5. Precursor dynamic exclusion was enabled with a duration of 30 s. Spectra were searched against the 2015 UniProt human databases (UP000005640_9606 and UP000005640_9606_additional) using MaxQuant version 1.5.3.34 with a 1% FDR at the peptide and protein level and default settings. The ‘match between runs’ feature was on with a matching time window of 0.7 min and an alignment time window of 20 min. Label‐free quantification was performed using the MaxLFQ algorithm with a minimum ratio count of 1. Following bioinformatic analyses were performed using the perseus software (Tyanova *et al.,* 2016) (version 1.5.5.0). Proteins that were identified in the decoy reverse database or only by site modification were removed for data analysis. The MS proteomics data have been deposited to the ProteomeXchange Consortium via the PRIDE (Vizcaino *et al.*, 2016) partner repository and are available upon request.

## 3 Results

### 3.1 Transcriptome profiling of DLD‐1 wt and DLD‐1 G13D cells

**Figure 1:**
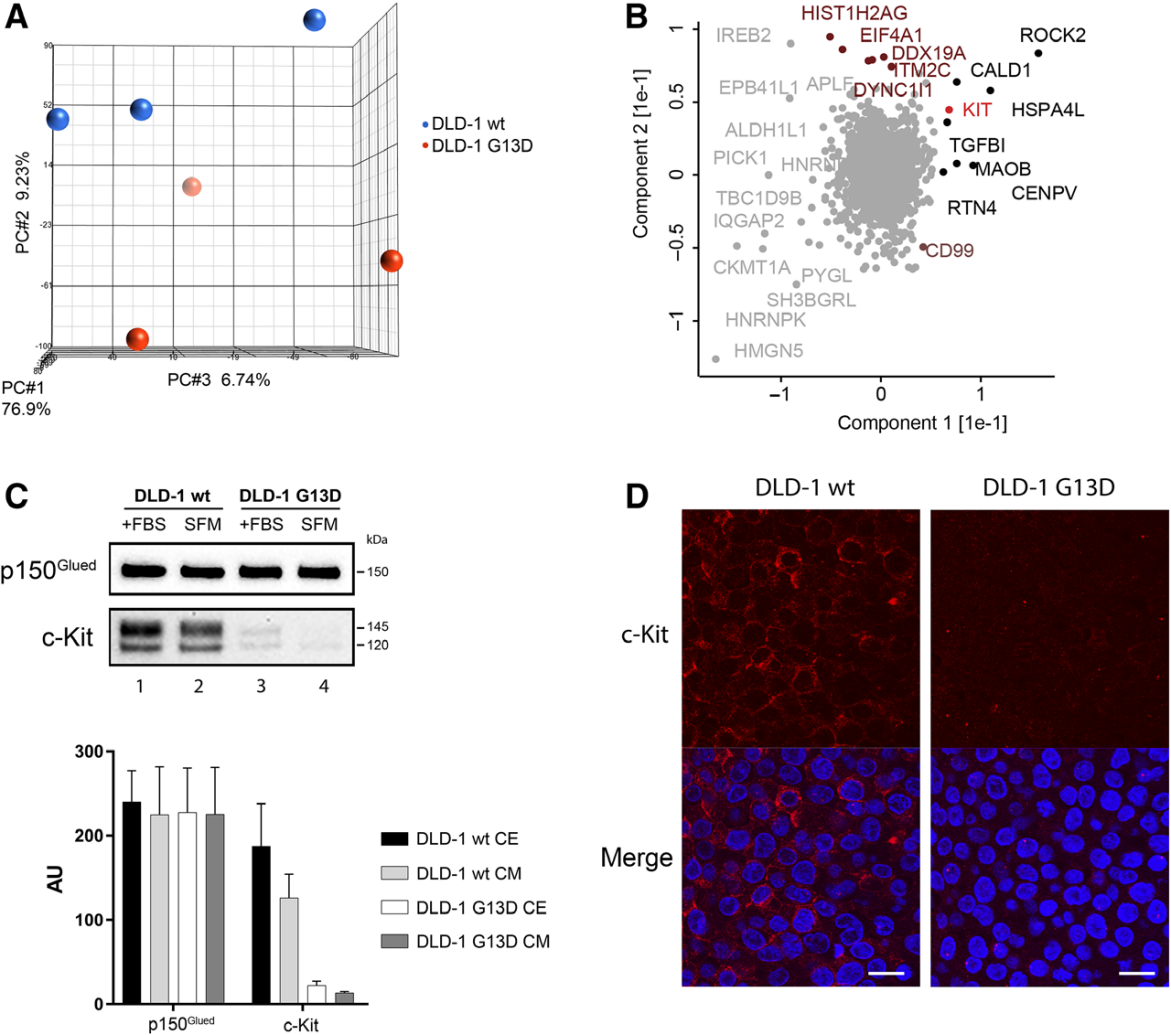
(A) PCA plot of expression microarray data of DLD‐1 wt (red circles) and DLD‐1 G13D (blue circles) cell lines. The percentages of the total variation that are accounted for by the 1st, 2nd, and 3rd principal components are shown on the *x*‐, *y*‐, and *z*‐axes labels, respectively. (B) PCA plot of comparative analysis between DLD‐1 wt and DLD‐1 G13 protein expression data, respectively. Proteins within the GO class ‘mitotic cell cycle’ are highlighted (magenta). c‐Kit is also pinpointed (red). (C) Upper panel: immunoblot analysis of c‐Kit and p150^Glued^ (normalizing control) protein expression in DLD‐1 wt and DLD‐1 G13D cells grown in media with serum (+FBS) and sereum free media (SFM), respectively, lower panel: graph depicting pooled densitometry measurements of c‐Kit levels relative to those of p150^Glued^in DLD‐1 wt and DLD‐1 G13D CE and CM, respectively. Data points are presented as mean ± SEM of triplicate experiments. (D) c‐Kit expression was examined in both DLD‐1 wt and DLD‐1 G13D cells by immunocytochemistry. Sections were immunofluorescently stained for c‐Kit (red) and the cell nucleus (DAPI, blue), respectively. Scale bar = 20 µm.

To address the molecular mechanisms underlying the differential invasive phenotype we had observed in *KRAS*‐mutated cells exposed to TIMP‐1 (Tarpgaard *et al.*, 2016), we performed genome‐wide mRNA expression profiling of the DLD‐1 wt and DLD‐1 G13D cell lines (dataset provided as Data S1). Given that the two cell lines are isogenic, only differing in their *KRAS* mutational status, we first determined whether we could find any biologically relevant linear dimensions in the expression dataset that would differentiate between the two cell lines. Principal component analysis (PCA) of our dataset showed that the two cell lines could be separated on the basis of their three main principal components (PC‐#1, PC‐#2, and PC‐#3, respectively; Fig. 1A). We then compared the two cell lines’ mRNA profiles and found only rather moderate differences in mRNA expression between the two lines. The 25 most highly up‐ or down‐regulated transcripts are presented in Table 1. Two differentially expressed genes (Table 1) called our attention: the *KIT* proto‐oncogene RTK (c‐Kit), a key regulator of cell survival, proliferation, and migration (Lennartsson and Rönnstrand, 2012), and tripartite motif‐containing 59 (TRIM59) protein, which is known to affect CRC proliferation and metastasis (Sun *et al.*, 2017). These two genes stood out, not only because they are known to affect CRC cell invasion, but also, importantly, because they were present in a set of 58 hits we discovered in a yeast two‐hybrid (Y2H) screening for TIMP‐1 potential interaction partners (M. Hoeberg, personal communication).

**Table 1:** List of top 25 genes, which showed differential expression between DLD‐1 wt and DLD‐1 G13D, ranked by *P*‐value, with cut off Log2 FoldChange>+/‐1.

	Gene		Log_2_FoldChange			*P*‐value		
	Symbol	Name	wt vs G13D	wt vs wt + TIMP‐1	G13D vs G13D + TIMP‐1	wt vs G13D	wt vs wt + TIMP‐1	G13D vs G13D + TIMP‐1
1	S100P	S100 calcium binding protein P	3.30	0.10	−0.40	0.00000	0.691	0.138
2	PDZK1IP1	PDZK1 interacting protein 1	−1.72	0.10	0.30	0.00001	0.657	0.209
3	SH3BGRL	SH3 domain binding glutamate‐rich protein‐like	1.50	−0.33	0.09	0.00003	0.168	0.699
4	KBTBD11	Kelch repeat and BTB (POZ) domain‐containing 11	1.68	0.09	−0.39	0.00004	0.742	0.172
5	HAGH	Hydroxyacylglutathione hydrolase	−1.74	−1.56	0.39	0.00006	0.000	0.198
6	KLK7	Kallikrein‐related peptidase 7	1.74	−0.17	−0.50	0.00006	0.558	0.104
7	NPPB	Natriuretic peptide B	1.90	0.12	0.49	0.00006	0.715	0.138
8	CYB5A	Cytochrome b5 type A (microsomal)	1.55	0.20	0.51	0.00008	0.465	0.075
9	TRIM59	Tripartite motif‐containing 59	−1.46	0.26	−0.43	0.00009	0.326	0.114
10	PPT2	palmitoyl‐protein thioesterase 2	1.82	0.47	−0.13	0.00018	0.188	0.714
11	KIT	v‐Kit Hardy–Zuckerman 4 feline sarcoma viral oncogene homolog	−1.33	0.33	−0.08	0.00025	0.221	0.771
12	KRT20	keratin 20, type I	1.99	0.35	−1.07	0.00026	0.376	0.017
13	GFRA4	GDNF family receptor alpha 4	−1.96	−1.68	0.20	0.00028	0.001	0.605
14	SEZ6L2	Seizure‐related 6 homolog (mouse)‐like 2	1.25	0.26	0.11	0.00029	0.320	0.651
15	ICAM2	intercellular adhesion molecule 2	−1.76	−0.07	0.73	0.00035	0.852	0.063
16	LKAAEAR1	LKAAEAR motif‐containing 1	−1.89	−1.50	0.38	0.00042	0.002	0.350
17	KIAA1211L	KIAA1211‐like	−1.28	−1.02	0.17	0.00046	0.002	0.541
18	B3GNT9	UDP‐GlcNAc:betaGal beta‐1,3‐N‐acetylglucosaminyltransferase 9	−1.23	0.11	0.39	0.00055	0.693	0.158
19	DENND2D	DENN/MADD domain‐containing 2D	1.21	0.01	0.07	0.00057	0.957	0.800
20	PDE4D	Phosphodiesterase 4D, cAMP‐specific	2.60	0.29	1.59	0.00057	0.615	0.015
21	FOXB1	Forkhead box B1	−1.58	−1.37	0.81	0.00059	0.002	0.035
22	LOXL1	Lysyl oxidase‐like 1	1.11	0.19	0.43	0.00066	0.447	0.100
23	LAGE3	L antigen family, member 3	1.75	0.49	1.06	0.00075	0.226	0.018
24	NMB	Neuromedin B	1.15	−0.12	−0.39	0.00086	0.654	0.159
25	TNNT1	Troponin T type 1 (skeletal, slow)	1.24	−0.14	0.58	0.00086	0.638	0.062

### 3.2 Proteome profiling of DLD‐1 wt and DLD‐1 G13D cells

To further address the molecular mechanisms underlying the TIMP‐1‐dependent differential invasive phenotype we observed in *KRAS*‐mutated cells, we performed MS‐based proteomic analysis of the DLD‐1 wt and DLD‐1 G13D cell lines. We quantified a total of 5861 proteins (Data S2). Again, PCA along the two main principal components segregated the two cell lines, with the main component driving the separation being proteins within the GO class ‘mitotic cell cycle’. This observation is consistent with the presence of the oncogenic *KRAS* G13D allele in DLD‐1 G13D cells, and a higher proliferative potential than DLD‐1 wt cells. Among the proteins identified as being differentially expressed between the two cell lines, we identified c‐Kit (Fig. 1B), which was concordant with our mRNA expression data (Table 1). TRIM59 was not present in our protein dataset, so we could not confirm its deregulation. We confirmed the decreased c‐Kit expression in DLD‐1 G13D cells compared to wt cells by western blot analysis of cellular extracts (CE; Fig. 1C) and immunofluorescence (Fig. 1D). As the extracellular domain of c‐Kit can be shed to the extracellular environment following proteolytic cleavage, it was conceivable that the difference in cellular expression we had observed was due to substantially increased protein shedding to the extracellular space, rather than true differential expression (Yee *et al.*, 1994; Yee *et al.*, 1993). Analysis of conditioned media (CM) showed this not to be the case (CM; Fig. 1C), as the amount of extracellular c‐Kit present in CM of DLD‐1 G13D cells was markedly lower than the levels observed in CM of DLD‐1 wt cells. In both cases, the extracellular expression of c‐Kit was proportional to the cellular levels of c‐Kit (Fig. 1C; compare c‐Kit in DLD‐1 wt CE to CM and DLD‐1 G13D CE to CM, respectively).

### 3.3 TIMP‐1 interacts with the cellular membrane in DLD‐1 wt and DLD‐1 G13D cells

**Figure 2:**
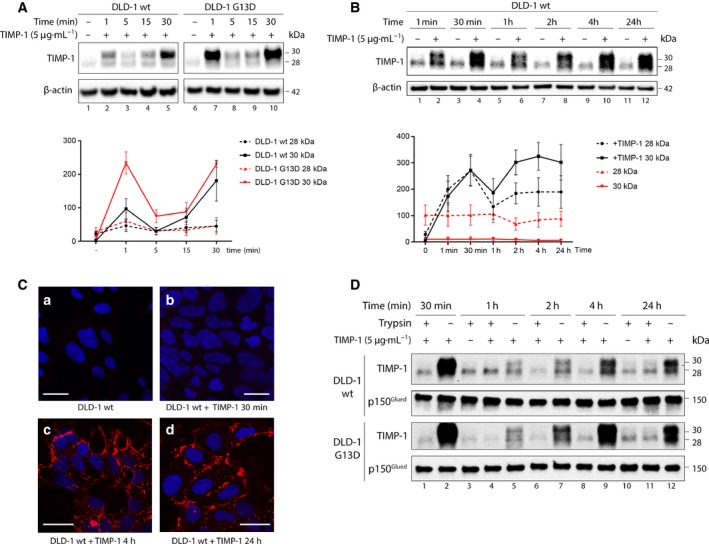
Presence and localization of exogenously added TIMP‐1 to DLD‐1 wt and G13D cells. (A, B) DLD‐1 wt and G13D cells were grown in the presence (or not) of TIMP‐1 (5 µg·mL^−1^) for the indicated amount of time. Cells were washed extensively, and whole‐cell extracts were examined by western blotting. Upper panels: immunoblots showing levels of TIMP‐1 protein present in whole‐cell extracts. β‐Actin was used as normalizing control. Lower panels: graphs depicting pooled densitometry measurements of TIMP‐1 levels relative to those of β‐actin. Data points are presented as mean ± SEM of triplicate experiments. (C) Immunostaining of DLD‐1 wt and G13D cells grown in the presence of TIMP‐1. Cells were grown in chamber slides and exposed to 5 µg·mL^−1^ TIMP‐1 for the indicated amount of time. Controls were grown in the absence of TIMP‐1. Cells were fixed and immunofluorescently stained for TIMP‐1 (red) and the cell nucleus (DAPI, blue), respectively. Scale bars, 20 µm. (D) DLD‐1 wt and G13D cells were grown in the presence of TIMP‐1 (5 µg·mL^−1^) for the indicated amount of time. Cells were washed extensively and trypsinized prior lysis and analysis of TIMP‐1 presence in whole‐cell extracts by western blotting. P150^Glued^ was used as normalizing control.

Previous studies indicated that the cellular effects of TIMP‐1 may be mediated by direct binding to a cell surface receptor, an assumption emphasized by the identification of the tetraspanin CD63 as a TIMP‐1 cell surface interacting protein in a complex with β1 integrin (Jung *et al.*, 2006; Toricelli *et al.*, 2013; Wilk *et al.*, 2013). Furthermore, uptake of TIMP‐1 in breast cancer cells has also been reported in co‐culture systems, suggesting that cancer cells can bind and internalize exogenous TIMP‐1 (Kuvaja *et al.*, 2012; Ritter *et al.*, 1999). We investigated whether DLD‐1 wt cells or DLD‐1 G13D cells can bind and internalize exogenously added human recombinant TIMP‐1 (Vinther *et al.*, 2014), and whether the profile of cellular internalization of TIMP‐1 differed between the two cell lines. TIMP‐1 was added to the culture media of DLD‐1 wt and DLD‐1 G13D cells, and samples were collected at different time points and analyzed (Fig. 2). We found that both DLD‐1 wt cells and DLD‐1 G13D cells could bind TIMP‐1, although the interaction differed between the two cell lines. In both cases, TIMP‐1 binding showed a bimodal behavior (Fig. 2A,B): a fast (1 min) but transient interaction in a first step, followed by a slower second step where we observed continuously increasing interaction of TIMP‐1 until a stable state was reached sometime between 2 and 4 h, indicating the binding/internalization process had reached equilibrium (Fig. 2B). The only detectable difference between the two cell lines concerned the first interaction, where levels of bound TIMP‐1 protein were significantly higher in DLD‐1 G13D than in DLD‐1 wt (Fig. 2A).

Given that this assay does not discriminate between TIMP‐1 bound to a receptor on the cell surface and protein that has been internalized, we performed immunolocalization of TIMP‐1. We found that exogenously added TIMP‐1 localized mainly to the membrane (Fig. 2C). Trypsinization of cells prior to analysis of interacting TIMP‐1 revealed that most of the exogenous TIMP‐1 protein that interacted with DLD‐1 G13D or DLD‐1 wt cells, respectively, could be removed by trypsinization of cells prior to analysis. These observations suggest that in the case of DLD‐1 G13D and DLD‐1 wt cells, exogenous TIMP‐1 could bind to the cell surface but was not internalized, at least at any detectable rate (Fig. 2D).

### 3.4 TIMP‐1 has different effects on DLD‐1 wt and DLD‐1 G13D cells

Having established a gene expression baseline for DLD‐1 wt and DLD‐1 G13D cells, we examined what changes took place upon cell exposure to exogenous TIMP‐1. DLD‐1 wt and DLD‐1 G13D cells were cultured in the presence, or absence, of TIMP‐1 (5 µg·mL^−1^) for 4 h, and their transcriptomes were profiled as before. For the analysis of the gene expression dataset, Log_2_FoldChange and statistical significance were calculated (*P* < 0.05, *t*‐test with correction for multiple hypothesis testing) (Benjamini and Hochberg, 1995). However, this analysis gave no significant hits, and we repeated the analysis with a lower stringency (*P* < 0.0075, *t*‐test with no multiple hypothesis correction). We found that only seven genes were significantly changed by TIMP‐1 exposure in DLD‐1 wt cells (Table 2), whereas 1183 genes were affected in DLD‐1 G13D cells (Table 3). Clearly, the extent of the overall effect of TIMP‐1 was much greater in the *KRAS*‐mutated cells than in the *KRAS* wt cells, and it was conceivable that the cumulative effect of the changes brought about by exposure to TIMP‐1 could account for the differential effect of TIMP‐1 on the KRAS‐mutated cells. Noteworthy, neither c‐Kit nor TRIM59 expression were affected by TIMP‐1 (Table 1). Interestingly, we found that five genes, which were significantly up‐regulated upon TIMP‐1 stimulation in DLD‐1 G13D cells but showed no change in DLD‐1 wt cells, mapped to the Ras‐Raf‐MEK‐Erk/JNK signaling axis (Table 4), indicating that TIMP‐1 may sustain increased activity in the Ras signaling axis in cells expressing oncogenic Ras, which may result in reinforced signaling through this pathway.

**Table 2:** Significantly deregulated genes in DLD‐1 wt cells exposed to TIMP‐1.

	Symbol	Name	Log_2_FoldChange	*P*‐value
1	HAGH	hydroxyacylglutathione hydrolase	−1.56	0.00016
2	GFRA4	GDNF family receptor alpha 4	−1.68	0.00091
3	FOXB1	forkhead box B1	−1.37	0.00176
4	LKAAEAR1	LKAAEAR motif‐containing 1	−1.50	0.00235
5	KIAA1211L	KIAA1211‐like	−1.02	0.00246
6	ARTN	Artemin	−2.21	0.00599
7	PTH2	parathyroid hormone 2	−1.03	0.00735

**Table 3:** List of top 25 genes, ranked by *P*‐value, affected by TIMP‐1 in DLD‐1 G13D cells.

	Symbol	Description	Log_2_FoldChange	*P*‐value
1	EVX1	Even‐skipped homeobox 1	−1.32	0.0018
2	STX10	Syntaxin 10	1.81	0.0020
3	ZNF692	Zinc finger protein 692	2.17	0.0020
4	MC1R	Melanocortin 1 receptor (alpha melanocyte‐stimulating hormone receptor)	1.91	0.0021
5	RASL10A	RAS‐like, family 10, member A	1.72	0.0021
6	LZTS2	Leucine zipper, putative tumor suppressor 2	1.60	0.0023
7	EREG	Epiregulin	−1.84	0.0024
8	FAM173A	Family with sequence similarity 173, member A	1.85	0.0025
9	PPIE	Peptidylprolyl isomerase E (cyclophilin E)	1.55	0.0026
10	CRYBA2	Crystallin, beta A2	2.11	0.0026
11	TCIRG1	T‐cell, immune regulator 1, ATPase, H + transporting, lysosomal V0 subunit A3	1.76	0.0028
12	GGA1	Golgi‐associated, gamma adaptin ear‐containing, ARF binding protein 1	2.07	0.0028
13	EDF1	Endothelial differentiation‐related factor 1	1.61	0.0028
14	GYLTL1B	Glycosyltransferase‐like 1B	1.87	0.0028
15	SQLE	Squalene epoxidase	−3.22	0.0028
16	TELO2	Telomere maintenance 2	2.09	0.0028
17	C14orf80	Chromosome 14 open reading frame 80	2.25	0.0029
18	WDR6	WD repeat domain 6	1.76	0.0029
19	CD151	CD151 molecule (Raph blood group)	2.05	0.0029
20	ASIC1	acid sensing (proton gated) ion channel 1	1.37	0.0029
21	DBET	D4Z4 binding element transcript (nonprotein coding)	1.93	0.0029
22	USP20	Ubiquitin specific peptidase 20	1.44	0.0029
23	RECQL4	RecQ protein‐like 4	1.63	0.0029
24	HMG20B	High mobility group 20B	1.84	0.0030
25	CKS2	CDC28 protein kinase regulatory subunit 2	‐2.61	0.0030

**Table 4:** Selected hits from the Ras‐Raf‐MEK‐Erk/JNK signaling axis significantly up‐regulated upon TIMP‐1 stimulation in DLD‐1 G13D cells.

	Log_2_FoldChange			*P*‐value		
Gene	wt vs G13D	wt vs wt + TIMP‐1	G13D vs G13D + TIMP‐1	wt vs G13D	wt vs wt + TIMP‐1	G13D vs G13D + TIMP‐1
RAF1	−0.05	0.01	1.93	0.9291	0.9834	0.0068
MAP3K1	−0.58	0.05	2.20	0.3576	0.9340	0.0036
MAP3K3	−0.21	−0.09	1.64	0.6709	0.8587	0.0056
MAP3K6	−0.40	0.03	2.10	0.5012	0.9612	0.0035
MAP4K2	0.18	0.22	1.63	0.7054	0.6521	0.0047

### 3.5 Kinome profiling of DLD‐1 wt and DLD‐1 G13D cells exposed to TIMP‐1

**Figure 3:**
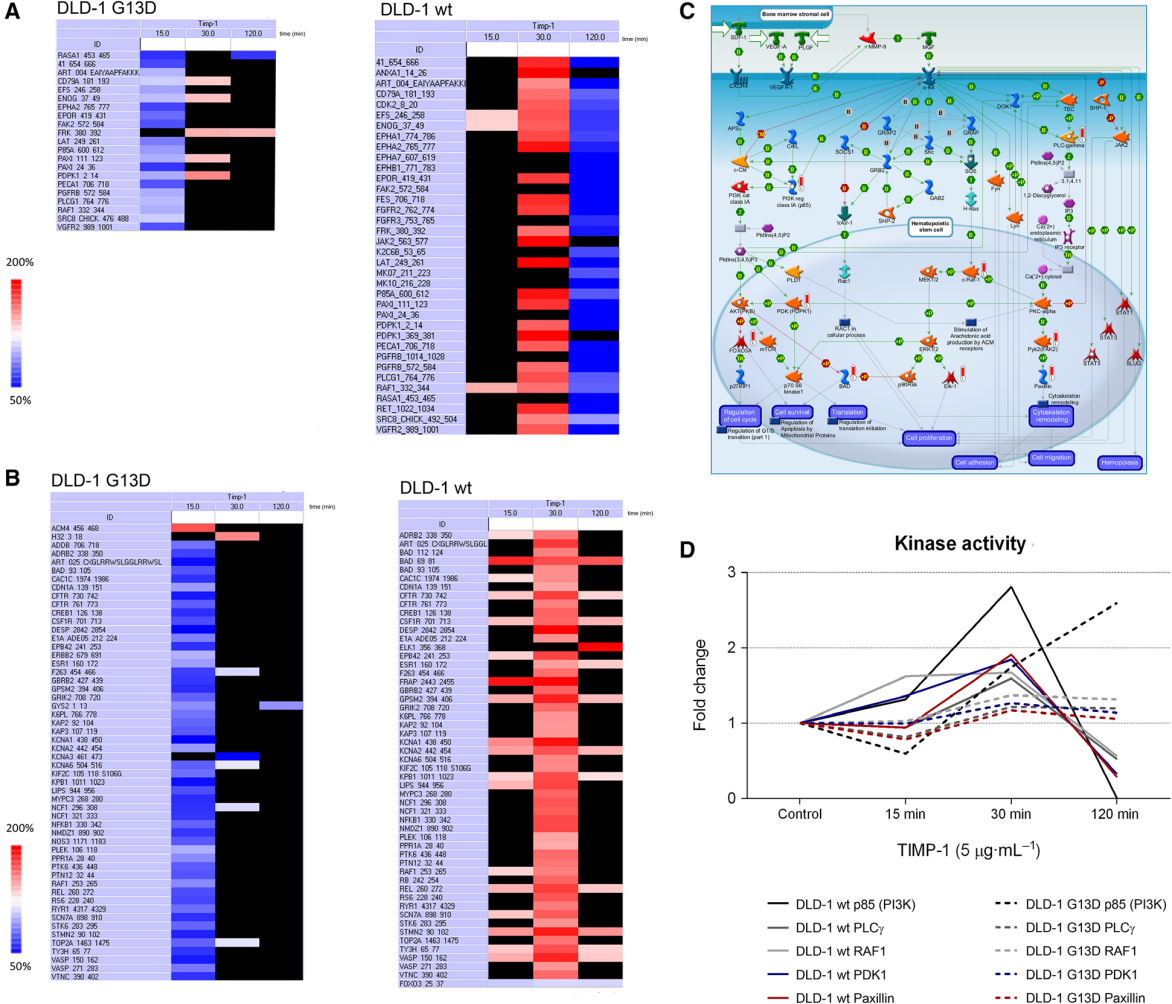
Kinase activity regulation results from PamGene analysis. DLD‐1 wt and DLD‐1 G13D cells were exposed (or not) with 5 μg·mL^−1^ TIMP‐1 for 15, 30, and 120 min and analyzed with PamGene kinase activity assay. The tables show significantly (*P* < 0.05) TIMP‐1‐regulated kinase activities of (A) protein TKs and (B) STKs (Red and blue are significant, *P* < 0.05; red denotes up‐regulation, and blue down‐regulation, respectively). Statistical tests were performed with PamGene’s Bionavigator software. (C) MetaCore pathway map visualization of PamGene kinase array data for the c‐Kit pathway. Kinases identified in our analysis are indicated by a histogram next to a network object with activity denoted by bar height (D) Analysis of kinase activity of kinases linked to the c‐Kit signaling axis upon exposure to TIMP‐1. Kinase activity raw data were extracted for c‐Kit‐related kinases (PI3K, PLCγ, RAF1, PDK1, and paxilin). The figure shows the mean fold change between the results of the control and TIMP‐1‐stimulated samples. Kinase activity was significantly down‐regulated upon 120 min in DLD‐1 wt, except for RAF1, which was borderline significant (*P* < 0.10).

TIMP‐1 has been shown to effect several distinct signaling pathways, including the mitogen‐activated protein kinase (MAPK) pathway and cAMP–protein kinase A pathway, and to be associated with activation of *RAS* and ERK, as well as PI3‐K induced Cyclin D expression. TIMP‐1 has also been reported to increase AKT activity (Akahane *et al.*, 2004; Lu *et al.*, 2011; Stetler‐Stevenson, 2008). Given that multiple associations with signaling pathways have been reported for TIMP‐1 and that these appear to be contextual, we performed a screening for kinases that may specifically be involved in TIMP‐1‐mediated CRC cellular signaling (Chirco *et al.*, 2006; Stetler‐Stevenson, 2008). Taking the patterns of association of exogenous TIMP‐1 with DLD‐1 G13D and wt cells in consideration (Fig. 2), we analyzed the kinome of DLD‐1 wt and DLD‐1 G13D cells, respectively, immediately before addition of exogenous TIMP‐1 to the growth medium, and after 15, 30, and 120 min of adding TIMP‐1 (Fig. 3D). Significant hits are reported in Fig. 3. TIMP‐1 stimulation had quite distinct effects on the two different cell lines (Fig. 3). For both TKs (Fig. 3A) and STKs (Fig. 3B), a general increase in kinase activity appeared in DLD‐1 wt cells exposed to exogenous TIMP‐1 for 30 min. For TKs only, a subsequent decrease was detected after 120 min compared to control cells (Fig. 3A). This pattern was different for DLD‐1 G13D, where significant inhibition of TK and STK activity, rather than activation, was observed upon 15 min of TIMP‐1 exposure followed by minor, nonsignificant, increases in TK activity after 30 and 120 min. This suggests *KRAS*‐dependent differences in TIMP‐1 signaling dynamics. Table 5 shows the top 10 hits for the ranking of active kinases derived from the STK and TK array analysis for A) protein TKs and B) STKs. Ranking is based on the sum of sensitivity (significance of the difference between samples) and specificity (specificity of the mapping to specific upstream kinases). One hit was particularly suggestive: c‐Kit, which we had found in our transcriptome and proteome profiling of DLD‐1 wt and DLD‐1 G13D cells to be up‐regulated in DLD‐1 wt cells compared to DLD‐1 G13D cells, both at the mRNA (Table 1) and at the protein levels (Fig. 1B), was on the list of kinases (Table 3). MetaCore pathway mapping of kinase hits, based on the enrichment distribution of the kinase dataset, identified the c‐Kit canonical signaling pathway as the highest significant [−log(*P*‐value) = 11] (Fig. 3C).

**Table 5:** Kinase analysis of TIMP‐1‐mediated effects.

Tyrosine kinases	Serine/threonine kinases
1 Insulin receptor (INSR)	1 Spleen tyrosine kinase (SYK)
2 Insulin‐like growth factor 1 receptor (IGF1R)	2 B lymphocyte kinase (BLK)
3 Insulin receptor‐related protein (INSRR)	3 Hepatocyte growth factor receptor (MET)
4 NT‐3 growth factor receptor (NTRK2)	4 Fibroblast growth factor receptor 3 (FGFR3)
5 High‐affinity nerve growth factor receptor (NTRK1)	5 Abelson murine leukemia viral oncogene homolog 1 (ABL)
6 v‐Kit Hardy–Zuckerman 4 feline sarcoma viral oncogene homolog (c‐Kit)	6 Receptor tyrosine‐protein kinase erbB‐3 (ERBB3)
7 Fibroblast growth factor receptor 4 (FGFR4)	7 Serine/arginine repetitive matrix protein 1 (SRMS)
8 Proto‐oncogene tyrosine‐protein kinase Src (SRC)	8 Gardner–Rasheed feline sarcoma viral (v‐fgr) oncogene homolog (FGR)
9 Tyrosine‐protein kinase Lyn (LYN)	9 Macrophage‐stimulating protein receptor (MST1R/RON)
10 Lymphocyte‐specific protein tyrosine kinase (LCK)	10 Related to receptor tyrosine kinase (RYK)

### 3.6 TIMP‐1 increases c‐Kit activity

Given that the Ras/MAPK and PI3K/Akt signaling modules are effectors of the c‐Kit signaling axis, and immunoprecipitation studies have shown that c‐Kit is associated with the tetraspanin CD63, a TIMP‐1 interactor, at the cellular membrane (Anzai *et al.*, 2002; Termini and Gillette, 2017), we investigated further a possible connection between c‐Kit and TIMP‐1. We performed co‐immunoprecipitation studies with c‐Kit and TIMP‐1, but were unable to detect any interaction, neither with a TIMP‐1‐specific antibody nor with a tagged version of TIMP‐1 (Fig. S1A,B, respectively), under the conditions assayed. PLA, a method that allows one to visualize protein–protein interactions in situ, allowed us to confirm that c‐Kit was in physical proximity of TIMP‐1, as we detected a number of fluorescent signals in DLD‐1 wt cells (Fig. S1C, panel a, white arrows), suggestive of an interaction between c‐Kit and TIMP‐1, whereas very few signals were detected in DLD‐1 G13D cells, which express c‐Kit at much lower levels (Fig. S1C, panel b, white arrows, and Fig. 1B).

**Figure 4:**
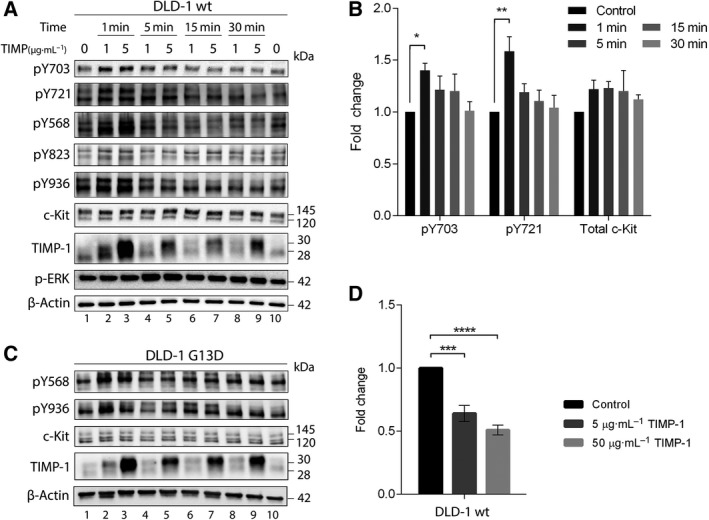
Phosphorylation of c‐Kit upon TIMP‐1 exposure. (A and C) DLD‐1 wt and G13D cells were stimulated with either 1 or 5 µg·mL^−1^ TIMP‐1 for the indicated time periods. Controls were treated with vehicle (PBS) for 30 min. Western blots were repeatedly stripped and probed for the different anti‐phospho‐c‐Kit antibodies recognizing various phosphorylated residues (Y703, Y721, Y568, Y823, and Y936 for DLD‐1 wt and Y568 and Y936 for DLD‐1 G13D, respectively) and finally for total c‐Kit. Blots were additionally blotted for pERK1/2 and TIMP‐1. β‐Actin was used as loading control. (B) Quantification of phosphorylated c‐Kit and total c‐Kit levels upon TIMP‐1 exposure in DLD‐1 wt cells. Quantification was performed with ImageJ. The mean fold change of c‐Kit pY703, c‐Kit pY721, and total c‐Kit levels between unstimulated and TIMP‐1‐treated samples is presented (±SEM). * indicates *P* < 0.05, and ** indicates *P* < 0.01, a two‐way ANOVA test with Dunnett’s correction for multiple comparison was used for significance. (D) Analysis of c‐Kit shedding upon exposure to TIMP‐1. Cells were stimulated for 24 h with 5 or 50 µg·mL^−1^ TIMP‐1 prior to collection of conditioned medium. Soluble c‐Kit levels in the medium were determined by a c‐Kit shedding‐specific ELISA kit. Bars represent the mean fold change of soluble c‐Kit levels between control and TIMP‐1‐stimulated samples (±SEM). No shed c‐Kit was detected for DLD‐1 G13D. *** and **** indicate *P* < 0.001 and *P* < 0.0001, respectively. Significance was determined by one‐way ANOVA test with Dunnett’s correction for multiple comparison.

Given that c‐Kit was among the top hits in our kinase analysis, we decided to investigate c‐Kit activity upon TIMP‐1 stimulation. c‐Kit phosphorylation was used as a measure of c‐Kit activity. Phosphorylation of all tested c‐Kit phosphorylation sites increased significantly upon exposure of DLD‐1 wt or DLD‐1 G13D cells to TIMP‐1 stimulation (Fig. 4A–D, respectively). To investigate whether TIMP‐1‐increased phosphorylation of c‐Kit is followed by phosphorylation downstream targets of c‐Kit, p‐ERK levels were also examined. We found increased (twofold) p‐ERK levels upon 5 min of TIMP‐1 stimulation in DLD‐1 wt (Fig. 4B).

**Figure 5:**
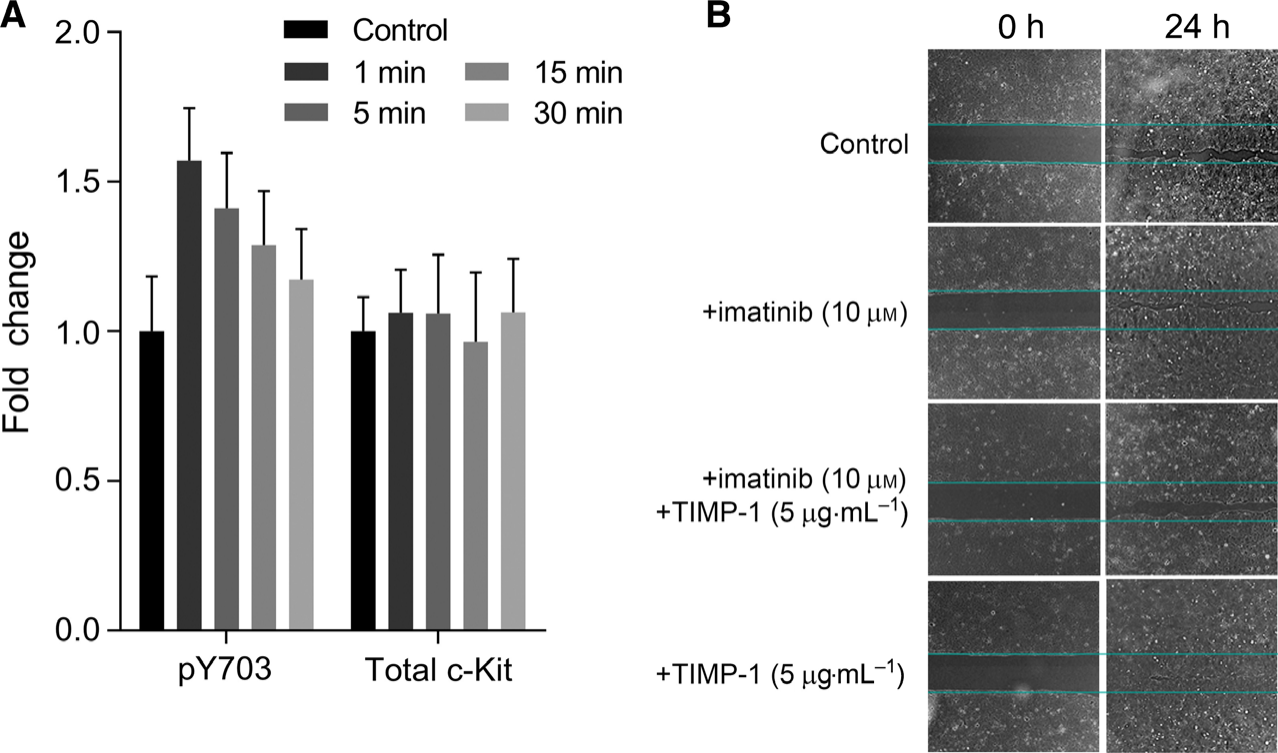
TIMP‐1 effects on (A) Ba/F3 pro‐B murine cells and (B) DLD‐1 wt cells. (A) Ba/F3 cells were stimulated with 5 µg·mL^−1^ TIMP‐1 for the indicated time periods. Samples were analyzed for phospho‐ (Y703) and total c‐Kit levels by western blot analysis. β‐Actin was used as loading control. Quantification was performed with imagej. The mean fold change of c‐Kit pY703, and total c‐Kit levels between unstimulated and TIMP‐1‐treated samples are presented (±SEM); a two‐way ANOVA test with Dunnett’s correction for multiple comparison was used for significance. (B) Light micrographs of wounded cell monolayers showing effect of TIMP‐1 on wound closure of DLD‐1 wt cells at 24 h. Cells were exposed to imatinib mesylate (10 µm), TIMP‐1 (5 µg·mL^−1^), or a combination of both. Representative images are shown.

Another level of regulation of c‐Kit function includes proteolytic extracellular cleavage allowing release of a surface protein ectodomain (‘shedding’) that produces a soluble form of c‐Kit. This reduces cell surface expression as well as produces a soluble c‐Kit polypeptide, which can competitively bind the c‐Kit ligand SCF. Stimulating DLD‐1 wt with either 5 or 50 μg·mL^−1^ TIMP‐1 significantly decreased soluble c‐Kit levels in the media down to ~ 60% and ~ 50%, respectively (Fig. 4D, black vs gray bars). These data are consistent with a previous observed decreased levels of soluble c‐Kit upon incubation of mast cells with TIMP‐1 and TIMP‐3 (Cruz *et al.*, 2004). To determine whether the effect of TIMP‐1 on c‐Kit activation we observed was a DLD‐1 wt cell‐specific effect, we examined levels of pY703 c‐Kit in Ba/F3 cells upon exposure to TIMP‐1. Ba/F3 is a murine, interleukin (IL)‐3 dependent, hematopoietic cell line (Palacios and Steinmetz, 1985). We found that TIMP‐1 induced activation of c‐Kit, measured by increased phosphorylation of pY703 (Fig. 5A).

## 4 Discussion

Several studies have reported that plasma levels of TIMP‐1 have prognostic value in CRC (Birgisson *et al.*, 2010; Nielsen *et al.*, 2010). Although TIMP‐1 expression is up‐regulated in colorectal tumor tissue (Lu *et al.*, 1991), the molecular mechanisms underlying the prognostic effect of TIMP‐1 in CRC remain unclear. We previously reported a differential cellular effect of TIMP‐1 on CRC cell invasion, dependent on *KRAS* status (Tarpgaard *et al.*, 2016). A similar observation has also been reported for pancreatic ductal adenocarcinoma, where exogenous addition of human recombinant TIMP‐1 significantly increased proliferation of pancreatic ductal cells, but only in *KRAS*‐transformed cells (Botta *et al.*, 2013). To gain some insight into the molecular mechanisms underlying the *KRAS*‐dependent effects of TIMP‐1 in CRC, we examined a matched pair of isogenic human CRC DLD‐1 cell line clones (*KRAS* G13D and wild‐type) exposed, or not, to TIMP‐1. We performed genome‐wide mRNA expression profiling of the DLD‐1 wt and DLD‐1 G13D cell lines (Table 1 and Fig. 1) and, from the limited number of differentially expressed genes between the two cell lines, identified two, c‐Kit and TRIM59, that could conceivably play a role in the effects of TIMP‐1 on CRC cells. These two genes stood out, not only because they are known to affect CRC cell invasion, but also, importantly, because they were present in a set of 58 hits we discovered in a Y2H screening for TIMP‐1 potential interaction partners (M. Hoeberg, personal communication). We complemented the gene expression analysis of the DLD‐1 wt and DLD‐1 G13D cell lines with MS‐based proteome profiling and phosphopeptide array‐based kinome profiling. We found that all levels of our analyses linked c‐Kit, expression, and pathway activation, with exposure of cells to TIMP‐1. In fact, targeted analysis of c‐Kit activation showed that TIMP‐1 significantly increased the phosphorylation of c‐Kit docking sites for GRB2 and P85 (pY703 and pY719) in DLD‐1 wt. GRB2 has a major role in being the adapter protein linking the scaffolding protein GAB2 to c‐Kit. Activation of c‐Kit leads to phosphorylation of GAB2 and the recruitment of SHP2, which leads to RAS/ERK pathway activation, as well as p85, which in turn leads to PI3‐K pathway activation (Sun *et al.*, 2008). In addition, the increased phosphorylation of c‐Kit observed in DLD‐1 G13D suggests that TIMP‐1 causes c‐Kit phosphorylation independently of *KRAS* mutational status, though the levels of phosphorylated c‐Kit still differed, presumably due to the difference in total c‐Kit levels. These results showed that TIMP‐1 increased phosphorylation, and therefore c‐Kit activity, as well as increased the activity of c‐Kit canonical targets (Fig. 4). Activation of c‐Kit by TIMP‐1 was not a cell‐line‐specific event, as we observed the same effect in Ba/F3 pro‐B murine cells (Fig. 5A). These observations raise two major questions, how does TIMP‐1 cause activation of c‐Kit and is there a biological significance for this activation?

Addressing the first question, protein interaction analysis, be it co‐immunoprecipitation or pull‐down assays, failed to show any physical interaction between TIMP‐1 and c‐Kit (Fig. S1A). We did detect a number of interactions using an in situ assay (Fig. S1B), but the number of interaction was very low compared to the presence of c‐Kit at the membrane (Fig. 1D) and the availability of TIMP‐1 (Fig. 2C). With the caveat of a very transient interaction between TIMP‐1 and c‐Kit, our data were suggestive of physical proximity rather than a direct interaction between these two proteins. TIMP‐1 interacts with a CD63/β1‐integrin receptor at the cell membrane (Jung *et al.*, 2006); c‐Kit is also associated with the tetraspanin CD63 (Anzai *et al.*, 2002). Tetraspanin‐enriched microdomains (TEM) are functional platforms, capable of regulating multiple cellular processes, including ectodomain ‘shedding’ and signaling (Termini and Gillette, 2017; Yanez‐Mo *et al.*, 2011). We could, in fact, detect a significant effect of TIMP‐1 on shedding of c‐Kit (Fig. 4D), supporting the idea that proximity of TIMP‐1 and c‐Kit mediated by association within a TEM may be responsible for the activation of c‐Kit we observed.

Concerning the second question, on whether there is a biological significance for the TIMP‐1‐mediated activation of c‐Kit, we investigated the ability of TIMP‐1 to stimulate migration and proliferation of DLD‐1 wt cells, using an *in vitro* wound healing assay (Fig. 5B). We found that wound closure in DLD‐1 wt cells stimulated with TIMP‐1 was complete (100% confluency in the wound area) whereas in DLD‐1 wt cells, where no TIMP‐1 was added or where c‐Kit signaling was inhibited wound closure was delayed (~ 75% confluency in the wound area). These data suggest a TIMP‐1‐mediated effect on cell migration and proliferation in c‐Kit‐expressing CRC cells. These data are congruent with results from a recent study that showed that a subpopulation of endogenous cardiac stem cells, which express c‐Kit and CD63 (CD63^+ve^/c‐Kit^+ve^), was driven to proliferate, express cardiogenic factors, and differentiate into major cardiac cell types upon stimulation with TIMP‐1 (Abdelli and Singla, 2015).

## 5 Conclusions

In conclusion, we identified c‐Kit as a potential mediator of TIMP‐1 function in CRC cells and showed that TIMP‐1 can mediate activation of c‐Kit and prevent ectodomain shedding. Given the growing attention that c‐Kit has been gaining as an important oncogene in CRC (Attoub *et al.*, 2002; Zhang *et al.*, 2014) and the role it plays in regulation of cell proliferation and migration, it is likely that some of the biological effects of TIMP‐1 overexpression in CRC may be exerted through its effect on c‐Kit signaling. However, the *KRAS*‐associated effect of TIMP‐1 on CRC cell invasion cannot be explained by its potentiation of c‐Kit signaling alone, as this took place independently of *RAS* status. We did find *KRAS* status‐associated differences between the two cell lines both in terms of association with TIMP‐1 where levels of bound TIMP‐1 protein were significantly higher in DLD‐1 G13D than in DLD‐1 wt (Fig. 2A), and in terms of expression of genes affected by TIMP‐1 where we found that only seven genes were significantly changed by TIMP‐1 exposure in DLD‐1 wt cells (Table 2), whereas 1183 genes were affected in DLD‐1 G13D cells (Table 3). Five genes in particular, which were significantly up‐regulated upon TIMP‐1 stimulation in DLD‐1 G13D cells but showed no change in DLD‐1 wt cells, were interesting as they all mapped to the Ras‐Raf‐MEK‐Erk/JNK signaling axis (Table 4). It is conceivable that TIMP‐1 may cause increased activity in the Ras signaling axis in cells expressing oncogenic Ras, resulting in persistent enhanced signaling through this pathway, which would explain the worse outcome we observed in CRC patients in association with TIMP‐1 expression (Tarpgaard *et al.*, 2016), but further studies will be needed to clarify these observations.

## Conflict of interest

The funding bodies played no role in the design of the study and collection, analysis, and interpretation of data as well as in writing of the manuscript. The authors declare no conflict of interest.

## Author contributions

SF, JS, LR, MM, and JMAM: Study design and conceptualization; CN, SD, ALSAM, MH, and JUK: Methodology and data acquisition; CN, SD, ALSAM, MH, JUK, and SF: Data analysis and interpretation; CN, SD, JS, LR, MM, and JMAM: Writing and editing the manuscript.

## Supporting information


**Fig. S1.** Interaction analysis of TIMP‐1 and c‐Kit. (A) DLD‐1 wt and DLD‐1 G13D cells were transfected with GFP or GFP‐TIMP‐1 24 h prior to lysis. Immunoprecipitation of lysates was performed with GFP‐TRAPs. TIMP‐1 and c‐Kit were examined in samples of untransfected cells (Ctrl), input (IN), unbound (UB), and immunoprecipitated fraction (IP) using western blot analysis. (B) DLD‐1 wt and DLD‐1 G13D cells were exposed to 5 µg·mL^−1^ TIMP‐1 or vehicle for 30 min prior to lysis. Co‐IP of lysates was performed with protein G sepharose and either mouse anti‐IgG control or VT4 (anti‐TIMP‐1 antibody). TIMP‐1 and c‐Kit were examined in input, unbound and immunoprecipitated (Bound) fraction using western blot analysis. P150Glued was examined to ensure true immunoprecipitation. The immunoprecipitation of TIMP‐1 was successful, however, c‐Kit was not detected in the immunoprecipitated fraction. (C) PLA of c‐Kit and TIMP‐1. DLD‐1 wt (a) and DLD‐1 G13D (b) cells were stimulated with 5 μg·mL^−1^ TIMP‐1 for 30 min prior to fixing and embedding. Embedded cell sections were examined for TIMP‐1 and c‐Kit proximity, detected as red dots by PLA assay, and cell nucleus were stained (DAPI, blue). (Representative pictures are shown, scale bars 20 μm).

**Table S1.** Antibodies used in this study.

**Data S1.** Gene expression data.

**Data S2.** Mass spectrometry proteomics data.
