# Supplementary figures and images for: Metallopeptidase inhibitor 1 (TIMP‐1) promotes receptor tyrosine kinase c‐Kit signaling in colorectal cancer
Source: Mol Oncol. 2019 Oct 24;13(12):2646–62. doi: 10.1002/1878-0261.12575 (PMC6887592; doi:10.1002/1878-0261.12575)

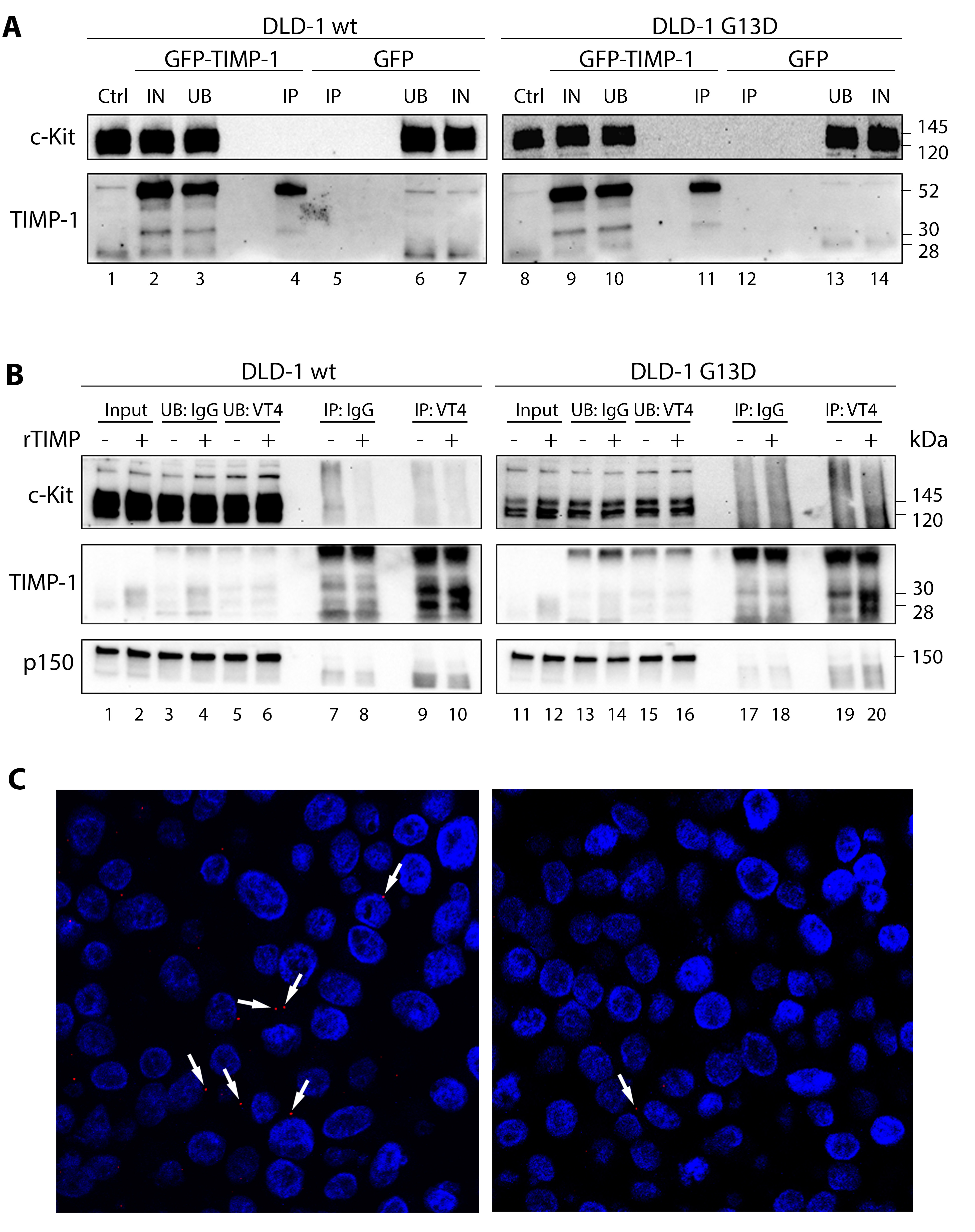

Supplement: Supplementary file 1 — Fig. S1. Interaction analysis of TIMP‐1 and c‐Kit. (A) DLD‐1 wt and DLD‐1 G13D cells were transfected with GFP or GFP‐TIMP‐1 24 h prior to lysis. Immunoprecipitation of lysates was performed with GFP‐TRAPs. TIMP‐1 and c‐Kit were examined in samples of untransfected cells (Ctrl), input (IN), unbound (UB), and immunoprecipitated fraction (IP) using western blot analysis. (B) DLD‐1 wt and DLD‐1 G13D cells were exposed to 5 µg·mL−1 TIMP‐1 or vehicle for 30 min prior to lysis. Co‐IP of lysates was performed with protein G sepharose and either mouse anti‐IgG control or VT4 (anti‐TIMP‐1 antibody). TIMP‐1 and c‐Kit were examined in input, unbound and immunoprecipitated (Bound) fraction using western blot analysis. P150Glued was examined to ensure true immunoprecipitation. The immunoprecipitation of TIMP‐1 was successful, however, c‐Kit was not detected in the immunoprecipitated fraction. (C) PLA of c‐Kit and TIMP‐1. DLD‐1 wt (a) and DLD‐1 G13D (b) cells were stimulated with 5 μg·mL−1 TIMP‐1 for 30 min prior to fixing and embedding. Embedded cell sections were examined for TIMP‐1 and c‐Kit proximity, detected as red dots by PLA assay, and cell nucleus were stained (DAPI, blue). (Representative pictures are shown, scale bars 20 μm). [file MOL2-13-2646-s001.jpg]
