# Supplementary material for: Metallopeptidase inhibitor 1 (TIMP‐1) promotes receptor tyrosine kinase c‐Kit signaling in colorectal cancer
Source: Mol Oncol. 2019 Oct 24;13(12):2646–62. doi: 10.1002/1878-0261.12575 (PMC6887592; doi:10.1002/1878-0261.12575)
Supplement: Supplementary file 2 — Table S1. Antibodies used in this study. [file MOL2-13-2646-s002.docx]

## Table S1. Antibodies used in this study

| Antigen | Species | Supplier | Clone | Catalog# | Dilution used |
| --- | --- | --- | --- | --- | --- |
| CD63 | Mouse mAb | EMD Millipore | NKI/C-3 | OP171 | 1:4000 |
| CD63 | Rabbit pAb | Santa Cruz Biotechnology | - | H-193 | 1:200 |
| CD74 | Rabbit pAb | Sigma-Aldrich | - | HPA010592 | 1:4000 |
| c-Kit | Rabbit pAb | provided by L. Rönnstrand | - | - | 1:1000 |
| c-Kit | Rabbit mAb | Cell Signaling Technology | D13A2 | 3074 | 1:1000 |
| c-Kit | Rabbit pAb | Dako | - | A4502 | 1:250 (ICC) |
| p150^Glued^ | Mouse mAb | BD Transduction Laboratories | Clone 1 | 610473 | 1:4000 |
| Phospho-PI3 Kinase p85 (Tyr458)/p55 (Tyr199) | Rabbit pAb | Cell Signaling Technology | - | 4228 | 1:1000 |
| Phospho-c-Kit (Tyr703) | Rabbit mAb | Cell Signaling Technology | D12E12 | 3073 | 1:750 |
| Phospho-c-Kit (Tyr719) | Rabbit pAb | Cell Signaling Technology | - | 3391 | 1:750 |
| Ras | Mouse mAb | BD Transduction Laboratories | Clone 18 | R02120 | 1:1500 |
| RhoGDI | Rabbit pAb | Cell Signaling Technology | - | 2564 | 1:1500 |
| β-Actin | Mouse mAb | Sigma-Aldrich | AC15 | A5541 | 1:200.000 |
| Timp-1 | Mouse mAb | From our own laboratory | VT4 | - | 1:2000 |
| Timp-1 | Mouse mAb | From our own laboratory | VT7 | - | 1:1000; 1:2000 - 1:4000 (ICC/IF) |
